# Surgical peripheral iridectomy via a clear-cornea phacoemulsification incision for pupillary block following cataract surgery in acute angle closure

**DOI:** 10.1186/s12886-018-0786-2

**Published:** 2018-05-18

**Authors:** Aiwu Fang, Peijuan Wang, Rui He, Jia Qu

**Affiliations:** Wenzhou Medical University Eye Hospital, Wenzhou, 325027 China

**Keywords:** Surgical peripheral iridectomy, Phacoemulsification, Pupillary block, Acute angle closure

## Abstract

**Background:**

To describe a technique of surgical peripheral iridectomy via a clear-cornea tunnel incision to prevent or treat pupillary block following phacoemulsification.

**Methods:**

Description of technique and retrospective description results in 20 eyes of 20 patients with acute angle closure with coexisting visually significant cataract undergoing phacoemulsification considered at risk of postoperative papillary block as well as two pseudo-phakic eyes with acute postoperative pupillary-block. Following phacoemulsification and insertion of an intraocular lens, a needle with a bent tip was inserted behind the iris through the corneal tunnel incision. A blunt iris repositor was introduced through the paracentesis and placed above the iris to exert posterior pressure and create a puncture. The size of the puncture was enlarged using scissors. For postoperative pupillary block the same technique was carried out through the existing incisions created for phacoemulsification.

**Results:**

Peripheral iridectomy was successfully created in all 22 eyes. At a mean follow-up of 18.77 ± 9.72 months, none of the iridectomies closed or required enlargement. Two eyes had mild intraoperative bleeding and one eye a small Descemet’s detachment that did not require intervention. No clinically significant complications were observed. Visual acuity and IOP improved or was maintained in all patients. The incidence of pupillary block in our hospital was 0.09% overall, 0.6% in diabetics and 3.5% in those with diabetic retinopathy.

**Conclusions:**

This technique of peripheral iridectomy via the cornea tunnel incision can be safely used during phacoemulsification in eyes at high risk of pupillary block or in the treatment of acute postoperative pupillary-block after cataract surgery. The technique is likely to be especially useful in brown iris, or if a laser is not available.

## Background

Pupillary block is a rare complication in cataract surgery wit IOL in lens bag [1]. Accordingly peripheral iridectomy (PI) is not performed during routine phacoemulsification but may be indicated in special situations such as implantation of an anterior chamber (or iris-fixated) lens and perhaps in patients prone to inflammation and pupillary block [1, 2].

Phacoemulsification is increasingly being used for the primary management of acute angle closure (AAC) [1, 3–7], where, theoretically the shorter axial length and higher risk of post-operative inflammation may increase the risk of postoperative pupillary block, especially if other risk factors like diabetes are present [1, 6, 8]. An elective intra-operative iridectomy is difficult to execute through the length of the corneal tunnel incision. The alternatives are to create an additional limbal incision for this purpose, or perform a laser peripheral iridotomy (LPI) either prior to or after surgery if required. In the setting of inflammatory post-operative pupillary block, LPI is more difficult to perform and is prone to occlusion. In this situation, it is even more difficult to perform LPI on Chinese people due to the brown irises, which are thicker than blue irises. Furthermore, a Neodymium-YAG laser is not universally available, especially in poorly resourced countries. We describe a surgical technique and retrospectively describe the results of performing a surgical iridectomy through the cornea tunnel incision used for phacoemulsification.

## Methods

This was a retrospective case series. The technique was used on a series of patients with AAC seen between September 2009 and January 2013 at the glaucoma unit of the eye hospital, Wenzhou Medical University. Patients were included if they had concomitant visually significant cataract where the surgeon considered the risk of pupillary block (AAC concomitant diabetic retinopathy, uveitis and short axial length) following phacoemulsification for AAC warranted an intra-operative iridectomy. The institutional review board approved the study and informed consent was obtained from all patients.

The inclusion criteria were: (1) AAC concomitant diabetic retinopathy, uveitis and short axial length (<22 mm); (2) visually significant cataract or pseudophakic eye following phacoemulsification recently; (3) IOP and inflammation was under control prior to surgery. Exclusion criteria were: (1) AAC was not caused by non-pupillary block glaucoma such as neovascular glaucoma, trauma; (2) corneal opacity prevent to perform a surgical iridectomy; (3) AAC with clear opening of laser peripheral iridotomy.

### Surgical procedure

**Fig. 1:**
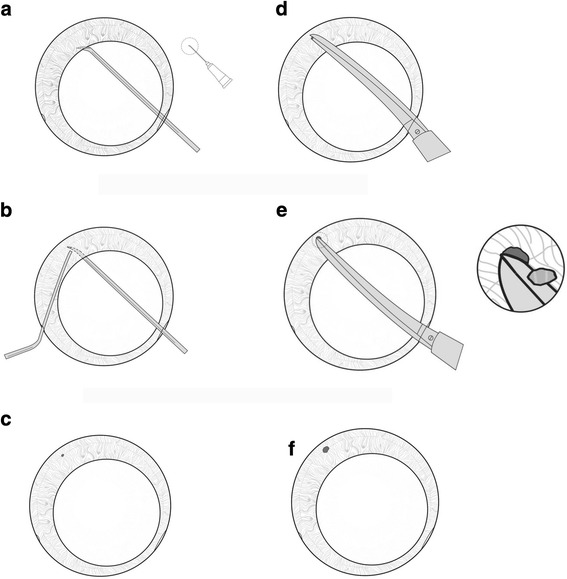
Steps of Procedure. **a.** 26G needle with its bent tip was kept horizontal to avoid snagging the iris. **b.** The needle tip was turned anteriorly towards the iris and assisted with the repository to create a puncture. **c.** A puncture was created. **d** and **e.** The puncture was enlarged by excising a small piece of iris tissue with scissors. **f.** The piece of iris tissue was removed by forceps and the iridectomy was finished

**Fig. 2:**
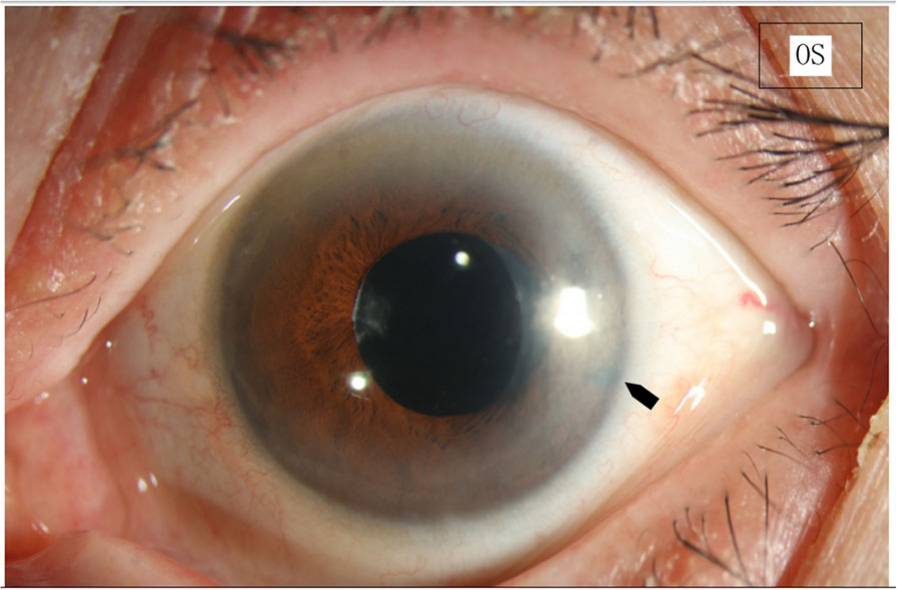
Patent Surgical Iridectomy**.** The iridectomy remained patent over a follow up of 12 months

Surgery was performed by a single surgeon under topical or peribulbar anesthesia. A standard clear-cornea phacoemulsification was performed with implantation of a foldable post chamber intraocular lens (IOL). The position of the clear-cornea tunnel was located in the nasal superior quadrant. Following insertion of the IOL in the capsular bag, a viscoelastic agent was injected into the anterior chamber and the posterior chamber at the site of the intended surgical iridectomy. A 26G needle with its tip bent to approximately 45 degrees about 1 mm from the tip was inserted into the corneal tunnel and advanced behind the iris, anterior to capsule and IOL to the selected site. The tip was kept horizontal to avoid snagging the iris. A blunt iris repositor was introduced into the eye through the paracentesis and placed above the iris adjacent to the needle. The needle tip was then turned anteriorly towards the iris and posterior pressure exerted with the repository to create a temperal inferior puncture. The size of the puncture was enlarged using fine long bladed microsurgical scissors to excise a small piece of iris tissue and the excised iris tissue removed with microsurgery forceps. The surgical technique can also create a nasal iridectomy through a temporal corneal tunnel. In the first three cases intra-cameral Carbamylcholine Chloride (0.01%) was administered to constrict the pupil following intraocular lens implantation. The surgical steps are illustrated in Fig. 1 and a typical post-operative result is shown in Fig. 2. Viscoelastic material was evacuated as is routine at the end of surgery.

The technique was used in twenty eyes of 20 patients with AAC and coexisting visually significant cataract considered to be at high risk of postoperative pupillary block due to the presence of factors such as poorly controlled diabetes, uveitis and short axial length. Two pseudophakic eyes that developed acute postoperative pupillary-block following phacoemusificatoin for AAC (in whom intraoperative iridectomy was not performed) also underwent this procedure.

### Clinical observations

Preoperative examination included decimal visual acuity (VA), applanation tonometry, biomicroscopy, ophthalmoscopy, gonioscopy, specular microscopy, and ultrasound biomicroscopy (UBM, OTI, Inc. CA). Anterior chamber depth was measured with UBM, Keratometry, and axial length were measured with an IOL Master (Carl Zeiss Meditec, Inc. Dublin, CA).

In all patients the acute attack was controlled with medications and/or paracentesis prior to surgery. Phacoemusification was undertaken as soon as the IOP decreased and the inflammation was under control. The time to surgery was 2 to 30 days.

Postoperative care included topical steroids and antibiotics, as is routine following phacoemulsification. Systemic steroids were administered if a fibrin reaction was seen in the anterior chamber. Patients were followed up on day 1 and 2, week 1 and 2, months 1, 2, 3, 6, 9, and 12 and then every 6 months thereafter. VA, intraocular pressure (IOP), disc assessment and postoperative complications were recorded.

### Statistical analysis

Statistical analysis was performed using the SPSS 20 software package (SPSS Inc., Munich, Germany). Results are reported as the mean ± standard deviation. A paired t test was used to evaluate changes in IOP and VA before and after surgery. VA was analyzed after conversion to the logarithm of the minimal angle resolution (logMAR) score. Hand-motion VA and counting fingers were assigned decimal equivalents of 0.001(+ 3.0 logMAR) and 0.01 (+ 2.0 logMAR). A *p* value less than 0.05 was considered statistically significant.

## Results

A patent surgical peripheral iridectomy was successfully achieved in the twenty eyes that underwent phacoemulsification for AAC and the two pseudophakic eyes with pupillary block that followed phacoemulsification.

Three patients were male and 19 were females. Mean patient age was 56.86 ± 12.90 years (range: 27 to 75). The table details demographics, eye characteristics and results. There were no complications during phacoemulsification and the IOL was implanted in capsular bag in all 20 eyes. During surgical peripheral iridectomy, two eyes had intraoperative bleeding resulting in a 1–2 mm hypaema that resolved spontaneously within a week. One eye had a small (approximately 1 mm^2^) Descemet’s detachment caused by the needle tip, but did not require any intervention. Two eyes that underwent phacoemulsification within two weeks of the acute attack developed intraoperative corneal edema that did not preclude completion of iridectomy. Complications such as iridodialysis/cyclodialysis were not observed and none of the patients reported dysphotopsia. All iridectomies remained patent through the mean follow-up of 18.8 ± 9.7 months (range 12–48 months).

Two patients had an IOP elevation on the first postoperative day. The IOP was lowered by pressure on the posterior lip of the paracentesis with a 26G needle at the slit lamp to allow egress of aqueous and any residual viscoelastic. None of the patients required long term anti-glaucoma medications.

Significant postoperative inflammation and corneal edema were the most common complications. Four eyes that developed a fibrin aqueous reaction and/or corneal edema had undergone surgery within 2 weeks of the acute attack. Three of the eyes had received intra-cameral Carbamylcholine Chloride; two eyes developed the fibrin reaction immediately after injection of this miotic. Four eyes developed significant stromal edema of the cornea, which subsided within 4 weeks.

Postoperative visual acuities improved or remained unchanged (Table 1). Visual acuity improved from 0.96 ± 1.03logMAR preoperatively to 0.18 ± 0.21 logMAR after surgery (*p* = 0.01).

**Table 1:** The demographics, eye characteristics and results of this study

Patient characteristics	Mean ± SD (range)	
Number of patients	22	
Female/male ratio	19/3	
Age	56.86 ± 12.90 (27–75)	
Follow-up range (months)	18.9 ± 9.72 (12–48)	
Diagnosis		
a. AAC with cataract (*n* = 20)		
PAS (clock hours)	6.95 ± 3.15 (0–12)	
ACD (mm)	1.59 ± 0.20 (1.22–1.90)	
AL (mm)	21.44 ± 0.58 (19.65–21.97)	
b. Postoperative pupillary block after phacoemulsification (*n* = 2)	Case 1	Case 2
PAS (clock hours)	9	11
ACD (mm)	3.75	3.15
AL (mm)	22.86	23.26
Preoperative data		
IOP at presentation (mmHg)	50.81 ± 6.01 (41–60)	
LogMAR BCVA	0.96 ± 1.03 (0.1–3)	
Postoperative data		
IOP at last follow up (mmHg)	12.95 ± 3.36 (8–20)	
LogMAR BCVA	0.18 ± 0.21 (0–0.8)	

*PAS* Periphery anterior aynechia, *ACD* Anterior chamber depth, *AL* Axial lenth, *IOP* Intraocular pressure, *BCVA* Best corrected visual acuity, *SD* Standard deviation

The mean preoperative IOP at presentation was 50.81 ± 6.0 mmHg (range, 41–60 mmHg) (Table 1). At the final examination for IOP was 12.95 ± 3.36 mmHg (range, 8–20 mmHg) without medication (Table 1). The difference in pre and post-operative IOP was statistically significant (*p* = 0.000).

## Discussion

Postoperative pupillary block is a rare complication following phacoemulsification [1]. A surgical iridectomy may be indicated in high risk situations such as implantation of an anterior chamber (AC) or iris-fixed intraocular lens, perhaps in patients who are more prone to inflammation such as diabetics and those with current and past inflammation, especially where a YAG laser is not available [1, 2, 8]. In eyes with posterior chamber IOL, papillary block may be related to excessive postoperative inflammation, with the formation of posterior synechiae [9, 10]. This risk is higher in diabetics [1, 8], and in angle closure glaucoma [11].

Phacoemulsification is increasingly being used for the primary management of AAC [1, 3–7]. Two randomized trials have investigated phacoemulsification as a primary treatment for AAC [3, 6]. None of the patients undergoing phacoemulsification in those studies developed pupillary block; as numbers were small the upper end of the confidence interval is actually compatible with a true rate of 10–22% [3, 6].

A short axial length and higher risk of post-operative inflammation may increase the risk of pupillary block [1, 2]. Gaton’s series of pupillary block following posterior IOL implantation included two patients (2/6) with known diabetic retinopathy and four (4/6) with glaucoma [1]. Diabetes was also present in 3 of 4 patients who developed acute postoperative pupillary-block following phacoemulsification [12]. Acute postoperative pupillary block occurred in 11 eyes (11 patients) of 12,016 phacoemulsifications (0.09%) performed by one unit in our hospital between 2011 and 2014 (unpublished data). 10 of the 11 patients that developed pupillary block were diabetic while one had uveitis. Pupillary block occurred in 10 of 1704 (0.6%) diabetics undergoing phacoemulsification; all had diabetic retinopathy. The incidence of pupillary block in those with diabetic retinopathy was 3.5% (10 of 290).

A surgical iridectomy may be warranted in cases considered high risk for postoperative pupillary block. Performing LPI postoperatively only if papillary block occurs is certainly an option. However LPI in the situation of postoperative pupillary block, especially in the presence of inflammation that follows an acute attack is more difficult and associated with more complications [13–16]. Moreover it is also more difficult to perform LPI in brown irises in this situation, and in many locations a YAG laser may not be available. If YAG laser is not available, the described technique can also be used for the pupillary block that may follow AC or iris fixated IOL’s.

Performing a surgical iridectomy through a tunnel incision can be technically challenging. An elegant technique that creates a small incision in the bed of a 4–5 mm scleral tunnel during the course of manual small incision cataract surgery has been described and can be used if a scleral tunnel is employed for phacoemulsification [17, 18]. This method can also be used with a corneal tunnel, but is easier with a longer incisional length and width. Also, an incision in the bed of a corneal tunnel has the potential to distort the wound and cause leakage; a watertight closure in this situation needs a larger tunnel length which can make phacoemulsification more difficult.

Other alternatives include an additional limbal incision for the iridectomy or the use of a vitrector. An extra limbal incision that requires a suture will be unattractive to most surgeons. We tend to avoid a vitrector primarily because it adds considerable expense to the procedure but also because it is more difficult and traumatic in a dilated pupil [19, 20].

Proper positioning of the iridectomy when performed through the tunnel is difficult in patients with the dilated pupils encountered in AAC. While miochol can be used to constrict the pupil, some pupils are unreactive, and, in our experience, inflammation in eyes that have recently suffered AAC is aggravated by Miochol [3, 6].

Potential complications of this method include complications related to introduction of a sharp needle behind the iris. While damage to the posterior capsule is conceivable, the needle is introduced under viscoelastic with the tip oriented horizontally and the maneuvers are undertaken following intraocular lens implantation. Introducing a scissor into the AC can cause damage to tissues. While an iridotomy alone might suffice, we elected to excise tissue, as the larger opening is less likely to occlude in the presence of inflammation. Bleeding from the iris and damage to Descemets membrane (as occurred in one case) and to the corneal endothelium is also possible. In order to avoid the immediate postoperative pressure spike, residual viscoelastic agent should be removed completely at the end of the surgery.

We acknowledge that an iridectomy adds an extra step to surgery and as there is potential for complications, it is only indicated in cases at higher risk of pupillary block. We also acknowledge that it is difficult to accurately predict post-operative pupillary block and that our own data suggests that some of the iridectomies were probably unnecessary. A risk of 3.5% has a number needed to treat of 29 and may help the decisions in specific situations/locations [21].

To conclude, this surgical technique for iridectomy can be safely and conveniently used for cases with or at high risk of postoperative pupillary block following cataract surgery, especially in settings where a YAG laser is not available.

## Abbreviations

AAC: Acute angle closure
AC: Anterior chamber
IOL: Intraocular lens
IOP: Intraocular pressure
logMAR: Logarithm of the minimal angle resolution
LPI: Laser peripheral iridotomy
PI: Peripheral iridectomy
UBM: Ultrasound biomicroscopy
VA: Visual acuity

## References

[1] Gaton DD, Mimouni K, Lusky M, Ehrlich R, Weinberger D (2003). Pupillary block following posterior chamber intraocular lens implantation in adults. Br J Ophthalmol.

[2] Stamper RL, Lieberman MF, Drake MV. Becker-Shaffer's diagnosis and therapy of the glaucomas. 7th ed. San Diego: Harcourt Publishers Limited; 2001.

[3] Husain R, Gazzard G, Aung T, Chen Y, Padmanabhan V, Oen FT, Seah SK, Hoh ST (2012). Initial management of acute primary angle closure: a randomized trial comparing phacoemulsification with laser peripheral iridotomy. Ophthalmology.

[4] Imaizumi M, Takaki Y, Yamashita H (2006). Phacoemulsification and intraocular lens implantation for acute angle closure not treated or previously treated by laser iridotomy. J Cataract Refract Surg.

[5] Hwang JU, Yoon YH, Kim DS, Kim JG (2006). Combined phacoemulsification foldable intraocular lens implantation, and 25-gauge transconjunctival sutureless vitrectomy. J Cataract Refract Surg.

[6] Lam DS, Leung DY, Tham CC, Li FC, Kwong YY, Chiu TY, Fan DS (2008). Randomized trial of early phacoemulsification versus peripheral Iridotomy to prevent intraocular pressure rise after acute primary angle closure. Ophthalmology.

[7] Teekhasaenee C, Ritch R (1999). Combined phacoemulsification and goniosynechialysis for uncontrolled chronic angle-closure glaucoma after acute angle-closure glaucoma. Ophthalmology.

[8] Naveh N, Wysenbeek Y, Solomon A, Melamed S, Blumenthal M (1991). Anterior capsule adherence to iris leading to pseudophakic pupillary block. Ophthalmic Surg.

[9] Ferris FL, Kassoff A, Bresnick GH, Bailey I (1982). New visual acuity charts for clinical research. Am J Ophthaloml.

[10] Vajpayee RB, Angra SK, Titiyal JS, Sharma YR, Chabbra VK (1991). Pseudophakic pupillary-block glaucoma in children. Am J Ophthalmol.

[11] Weinreb RN, Wasserstrom JP, Forman JS, Ritch R (1986). Pseudophakic papillary block with angle-closure glaucoma in diabetic patients. Am J Ophthalmol.

[12] Khor WB, Perera S, Jap A, Ho CL, Hoh ST (2009). Anterior segment imaging in the management of postoperative fibrin pupillary-block glaucoma. J Cataract Refract Surg.

[13] Sihota R, Lakshmaiah NC, Walia KB, Sharma S, Pailoor J, Agarwal HC (2001). The trabecular meshwork in acute and chronic angle closure glaucoma. Indian J Ophthalmol.

[14] Saw SM, Gazzard G, Friedman DS (2003). Interventions for angle-closure glaucoma: an evidence-based update. Ophthalmology.

[15] Sakai H, Ishikawa H, Shinzato M, Nakamura Y, Sakai M, Sawaguchi S (2003). Prevalence of ciliochoroidal effusion after prophylactic laser iridotomy. Am J Ophthalmol.

[16] Athanasiadis Y, de Wit DW, Nithyanandrajah GA, Patel A, Sharma A (2010). Neodymium: YAG laser peripheral iridotomy as a possible cause of zonular dehiscence during phacoemulsification cataract surgery. Eye (Lond).

[17] Blumenthal M, Kahana M (1997). Performing peripheral Iridectomy via a scleral tunnel incision: a new technique. Ophthalmic Surg and Lasers.

[18] Thomas R, Parikh R, Muliyil J (2003). Comparison between phacoemulsification and the Blumenthal technique of manual small-incision cataract surgery combined with trabeculectomy. J Glaucoma.

[19] Bitrian E, Caprioli J (2010). Pars plana anterior vitrectomy, hyaloido-zonulectomy, and iridectomy for aqueous humor misdirection. Am J Ophthalmol.

[20] Debrouwere V, Stalmans P, Van Calster J, Spileers W, Zeyen T, Stalmans I (2012). Outcomes of different management options for malignant glaucoma: a retrospective study. Graefes Arch Clin Exp Ophthalmol.

[21] Thomas R, Padma P, Braganza A, Muliyil J (1996). Assessment of clinical significance: the number needed to treat. Indian J Ophthalmol.

